# Tasks Ahead in Public Health
*Inaugural address at the installation of Professor Parry as President of the Society of Medical Officers of Health, October 14th, 1948.


**Published:** 1948

**Authors:** R. H. Parry

**Affiliations:** Medical Officer of Health for Bristol


### TASKS AHEAD IN PUBLIC HEALTH*

BY

Professor R. H. Parry, M.D., F.R.C.P., D.P.H.

*Medical Officer of Health for Bristol*

The last two years have been full of interest for those who are engaged in the study and practice of public health. Our thoughts have been taken back to the promoters of the Public Health Act of 1848, to the conditions that existed in those days and to the means that were adopted to try and improve those conditions. We have paid our tributes to the great pioneers—the first medical officers of health not only of Liverpool and of London, but of our own towns and districts. We have learnt to appreciate their difficulties and the qualities which they displayed in their struggles for better conditions.

Our attention, however, has not been allowed to dwell unduly on the past; for the National Health Service Act has projected our thoughts to the future. It is most important that those already in the service, as well as those who are about to enter it, should fully appreciate the changes and the difficulties that lie ahead. I shall try, without paying undue attention to the difficulties, to make a few constructive suggestions.

There are many fields of medical activity in which the medical officer of health will still continue to play a prominent part. He will continue as medical adviser to his local authority when exercising its responsibility for housing, sanitation, welfare services, and deprived children. He himself may or may not have administrative responsibility for these services. If he is not responsible for them he will be the freer to express his opinion and to give advice. Elsewhere[1] I have discussed fully our hopes with respect to the development of health centres and the future of the clinical services of the local authority. The medical officer of health may have considerable influence in regard to these matters, though effective control over them has passed to his colleagues on the executive council.

Henceforth the main task before the medical officer of health will be to keep himself acquainted with the health conditions in his area and to advise the local health authority thereon with a view to securing an amelioration of conditions affecting the health of the people of his district. In this matter he will have a direct responsibility to the people. This will be both a privilege and a great responsibility,

* Inaugural address at the installation of Professor Parry as President of the Society of Medical Officers of Health, October 14th, 1948.

and he will require tact, firmness and conviction to help him to carry out his task. He must be able to give the best possible advice to his authority and this he must do without fear or favour. It is not easy to combine the duties of administrator and adviser ; the relief from administrative responsibility recently given to the medical officer of health by the transfer of hospitals and in other ways should make his task as an adviser easier. In view of the great personal responsibility that will be put on the health officer in this matter, would it not be the right and proper course for him to receive the help and support of an advisory committee ? I am convinced of the advantages of such an arrangement.

Representatives of the public press should have access to the reports and the discussions of this committee. This is a matter of first-rate importance in which the interest of the people of the area should be the primary consideration.

There is a great difference between conditions in this country to-day and those that obtained in the days of Edwin Chadwick and John Simon. So we must not be unduly influenced by arguments used by them and their contemporaries for the purpose of creating an executive machinery for sanitary reform. Would those pioneers consider to-day, for example, that a medical officer of health should be " independent of private practice ? " The expression " independent of private practice " does not convey the same meaning in 1948 as it did when Chadwick wrote his famous report more than a hundred years ago. Indeed, to-day there are many reasons why the medical officer of health should be actively engaged as a private practitioner of social medicine or as a specialist epidemiologist, in each case with opportunity for research : the two activities are complementary. A public health medical officer suffers a serious disadvantage in not having the opportunity to visit the home and the family (except as a sanitary inspector). There is in consequence a risk of his becoming an " office-desk " specialist—of which we have not a few in the profession at the present time. Opportunities for the medical officer of health to make frequent visits to the homes are essential in order that he can become fully aware of the problems that arise there and can make a special study of them. The Royal Commission of 1869–71 considered that the poor law medical officers of those days had this opportunity—" the advantage, namely, of their daily familiarity with current facts as to ailments and local circumstances . . . which gave them an initial advantage towards becoming efficient officers of health." In my opinion this question and its implications, in the light of conditions to-day, are worth studying anew.

In a recent letter to *The Times* George Bernard Shaw suggested a select committee " to settle our political nomenclature ".

In our branch of medicine there is a good case for some investigation to clear up the nomenclature. Our predecessors were quite satisfied with the term "public health" and they did not object when "preventive medicine" appeared and focused their attention on the need to do all that could be done to prevent the ravages of disease. In social medicine an attempt is made to focus the attention of clinicians on the need for paying more attention to the aetiology of disease.

Professor Ryle is a strong advocate of this discipline—I quote his words :

"It is curious that aetiology in its wider sense should have so far lost the interest which it had for the older physicians.[2] Man, as a person and a member of a family and of much larger social groups, with his health and sickness intimately bound up with the conditions of his life and work—in the home, the mine, the factory, the shop, at sea, or on the land—and with his economic opportunity, has been inadequately considered in this period by the clinical teacher and hospital research worker."

The conception of social medicine should bring clinicians and public health workers much closer together. In any good team there must be some degree of overlapping, otherwise there will be a gap. Clinicians may wish to have direct information regarding environmental conditions to which their cases have been exposed. Public health workers should encourage this step forward by clinicians. On the other hand, public health workers may need to see how far preventive measures have been evaded or have been ineffective. I have no doubt but that clinicians would welcome this. All members of the team should meet at the case conference from time to time. This procedure is most desirable not only in the interest of research but also in the interest of the patient—for the better understanding of the causes of the disease. At university centres where undergraduate training takes place there is a particularly strong case for "socio-medical case conferences" as established at Oxford by Professor Ryle. But such conferences would no doubt be welcomed by medical officers of health in other centres in the interest of team training and for the benefit of the patient.

This is the time in our history when every medical officer of health should consider anew his duties and responsibilities. I have given but a few examples of opportunities available to us to explore new channels. Let those that are satisfied sit at the office desk and administer, but for the pioneering spirit there is still the open road.

REFERENCES

[1] "Some Thoughts on the Future of Public Health and of the M.O.H." *Public Health*, March, 1948. By Dr. R. H. Parry.

"The Public Health Service as a Career" *The Medical Officer*, Sept. 25th, 1948. By Dr. R. H. Parry.

[2] *Changing Disciplines*. By John A. Ryle.

